# Gallbladder volvulus: A case report and review of the literature

**DOI:** 10.1016/j.ijscr.2019.02.025

**Published:** 2019-02-27

**Authors:** Waad Farhat, Mohamed Ben Mabrouk, Houssem Ammar, Abdkader Mizouni, Mohamed Amine Said, Sami Lagha, Yesser ben cheikh, Rahul Gupta, Makram Moussa, Ali Ben Ali

**Affiliations:** a Department of General and Digestive Surgery, Hopital Sahloul, Sousse, Tunisia; b Department of Radiology, Hopital Sahloul, Sousse, Tunisia; c Department of Gastrointestinal Surgery, Synergy Institute of Medical Sciences, Dehradun, India

**Keywords:** GV, gallbladder volvulus, CT, computed tomography, Gallbladder volvulus, Acute cholecystitis, Cholecystectomy

## Abstract

Introduction: Gallbladder volvulus (GV) is a rare disease with less than 400 cases reported in the English literature. The pre-operative diagnosis of GV is difficult as none of the imaging modalities are accurate. Once diagnosed, the mainstay of treatment is emergency surgical derotation and cholecystectomy.

Presentation of case: A 83-old lady presented with right upper quadrant pain and fever for 3 days. Abdominal imaging revealed the presence of a distended, floating gallbladder located outside its normal fossa with thickened non-enhancing wall and a twisted pedicle suggestive GV. The patient underwent emergency laparotomy because the laparoscopic approach was refused by the anesthetist due to the history of pulmonary emphysema. Intraoperatively, the gallbladder was found be gangrenous and rotated in anti-clockwise direction around the cystic pedicle. The gallbladder was de-rotated followed by cholecystectomy.

Discussion: GV is an uncommon cause for abdominal pain and occurs due to rotation of gall bladder on its mesentery along the axis of the cystic duct and the cystic artery. Pre-operative diagnosis continues to be a major challenge with only 4 cases reported in the literature diagnosed with pre-operative imaging. This case is one of the rare cases diagnosed with pre-operative imaging. It is often misdiagnosed as acute cholecystitis before surgery. However, critical constellation of presenting signs and symptoms along with radiological findings may guide the surgeon to accurate and timely diagnosis of GV before surgical intervention.

Conclusion: Although rare, it is important to consider GV as a differential diagnosis in an elderly patient with acute cholecystitis.

## Highlights

• Gallbladder volvulus (GV) is a rare disease with less than 400 cases reported in the English literature.
• Preoperative diagnosis is a major challenge with only 4 cases being diagnosed on radiology.
• Critical constellation of presenting symptoms and signs along with radiology can help in timely diagnosis of GV.

## 1 Introduction

Gallbladder volvulus (GV) is a rare disease and its incidence remains undetermined, with less than 400 cases previously reported in the literature [1]. It mainly occurs in elderly woman, with clinic-radiological findings mimicking acute cholecystitis [2]. It is a surgical emergency, and the diagnosis is usually made intraoperatively [3]. We report a rare case of gallbladder volvulus in elderly female diagnosed preoperatively and review the literature to discuss the clinical and radiological aspects of this rare complication. This case has been reported in line with the SCARE criteria [4].

## 2 Case report

A 83-old lady, a known case of pulmonary emphysema, presented to our hospital with right upper quadrant pain evolving since 3 days. On clinical examination, the patient was febrile, hemodynamically stable, with tenderness in the right upper quadrant and a palpable tender gallbladder. Laboratory tests showed leukocytosis (WBC-16000 cell/ mm3) and liver function tests were normal.

**Fig. 1:**
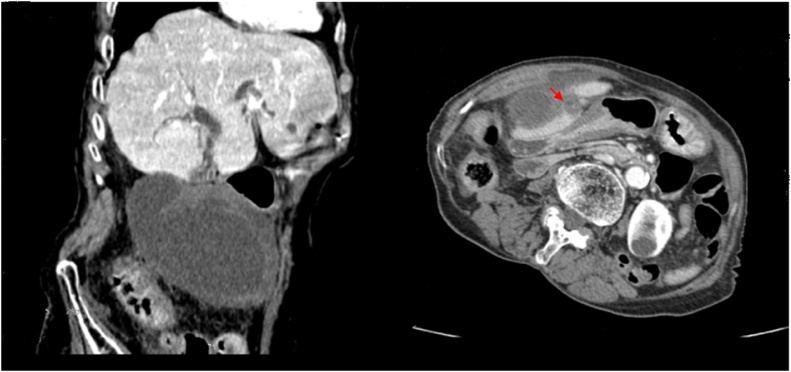
Abdominal CT revealed a distended, floating gallbladder located outside its normal fossa, with thickened wall and twisted pedicule (red arrow).

The abdominal ultrasonography revealed a distended gallbladder with thickened edematous wall and surrounding loculated ascites. No gallstones were seen and the intrahepatic and extrahepatic bile duct were not dilated. Abdominal computed tomography (CT) revealed the presence of a distended, floating gallbladder measuring 12.2 × 8.2 × 7.6 cm located outside its normal fossa with thickened non-enhancing wall and a twisted pedicle (Fig. 1).

**Fig. 2:**
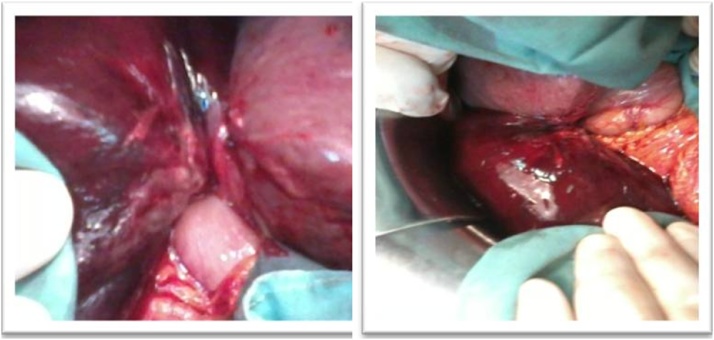
Intraoperative photo confirming the diagnosis of gangrenous cholecystitis complicating gallbladder volvulus.

**Fig. 3:**
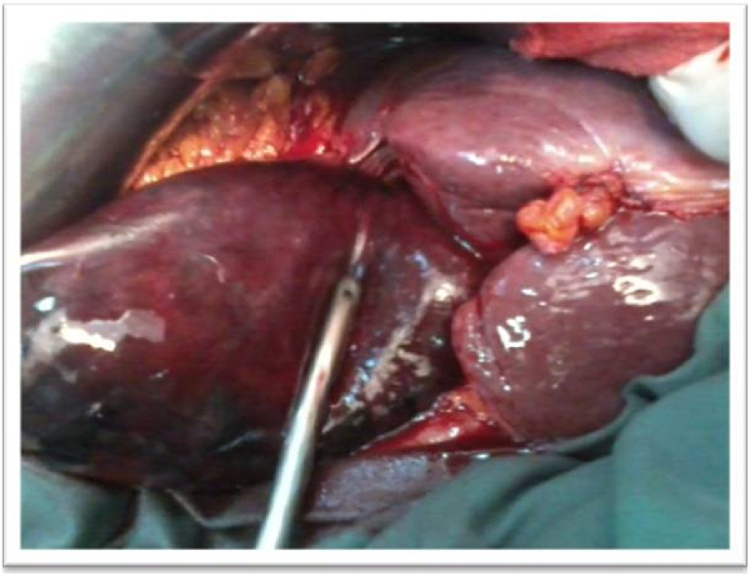
Intraoperative photo of distended and necrotic gallbladder after detorsion.

The diagnosis of acute cholecystitis complicating gallbladder volvulus was made. Intravenous

fluid, broad spectrum antibiotics and analgesics were administrated followed by emergency laparotomy via right subcostal incision, the laparoscopic approach was refused by the anesthetist due to the history of pulmonary emphysema. At surgery, a distended gangrenous gallbladder was found. The gallbladder was completely rotated anticlockwise (360°) around the cystic artery and the cystic duct (Fig. 2). After untwisting, it was found that the gallbladder had a complete long mesentery held closely to the liver (Fig. 3). Cholecystectomy was performed and suction drain was placed in the right subhepatic space. The postoperative course was uneventful. Histology revealed transmural gallbladder necrosis.

## 3 Discussion

The volvulus of gallbladder was first described by Wendel in 1898 as a floating gallbladder [5]. The clinical incidence of GV has been reported to be 1 in 365,520 hospital admissions and 85% percent of cases occur between the ages of 60 and 80 years, with a female-to-male ratio of 3:1 [2,6]. Some rare cases have been described in the pediatric population as early as 2 years of age [7].

GV is characterized by mechanical clockwise or counter clockwise organo-axial torsion along the longitudinal axis of the gallbladder involving cystic artery and cystic duct.

There are 5 recognized position of the gallbladder in relation to the liver: 1) intrahepatic; 2) closely attached to the liver surface by the peritoneum; 3) a complete mesentery but held closely to the liver; 4) a complete long mesentery that allows gallbladder to hang freely; 5) an incomplete mesentery which is attached along the cystic duct that allows gallbladder to hang freely in the peritoneum cavity. Only situation 4 and 5 can predispose to torsion [5,8].

Though the variation in peritoneal attachment of the gallbladder is congenital, the predisposing factor that are more commonly acquired include age > 70 years, female sex, weight loss, liver atrophy, kyphoscoliosis, and loss of visceral fat which results in the elongated gallbladder mesentery necessary for torsion to occur [5,6].

The importance of gallstones is unknown as approximately 70–80% of patients with gallbladder torsion had no gallstones. One study of 245 patients found stones in only 24.4% patients [9].

The inciting events of torsion may be either mechanical or hormonal changes that affect the gallbladder. The mechanical events may be sudden shifts in body position, intense peristalsis of adjacent viscera, and blunt trauma. Increased cholecystokinin production leading to gallbladder peristalsis after a fatty meal may facilitate gallbladder torsion [8].

The clinical features of gallbladder torsion are similar to those of acute cholecystitis. A low frequency of fever and jaundice, poor response to antibiotic therapy, and acute onset of abdominal pain may be helpful in the differentiating GV from acute cholecystitis and cholangitis [1,8].

Lau et al. has described a triad that is suggestive of GV [5]:

• Patient’s characteristics: thin, old patients with chronic lung disease or a spinal deformity.
• Symptoms: abdominal pain, short duration, early onset of vomiting.
• Signs: abdominal mass, lack of toxemia or jaundice, discrepancy in the pulse and the temperature.

Despite the technological advances in various imaging modalities, the pre-operative diagnosis of GV is very challenging with only 4 cases reported in the literature diagnosed with pre-operative imaging, the remaining cases were found intra-operatively [5,7]. This case is one of the rare cases diagnosed with pre-operative imaging. Abdominal ultrasound and CT scan often reveal a large floating gallbladder without gallstones and a thickened gallbladder wall. Specific signs seen with GV include the presence of the gallbladder outside its normal anatomic fossa, inferior to the liver or in a transverse orientation with an echogenic conical structure corresponding to the twisted pedicle. Magnetic resonance imaging (MRI) and magnetic resonance cholangiopancreatography (MRCP) can demonstrate necrosis or infarction or both while hepatobiliary iminodiacetic acid (HIDA) scans of GV are reported to resemble a bull’s-eye because accumulation of radioactive tracer within the gallbladder [2,3].

Once diagnosed, the appropriate treatment is emergency derotation and cholecystectomy. This can be performed by laparoscopy, which was first performed by Schroder and Cusumano in 1994, or by open technique [5]. With experience in laparoscopic cholecystectomy, laparoscopic derotation and cholecystectomy has become the preferred approach. If treated laparoscopically, gallbladder decompression and detorsion prior to cholecystectomy are helpful techniques to avoid bile duct injury [8]. However, due to medical co-morbidities, we did not attempt laparoscopy in the present case.

Prognosis is excellent if diagnosed and treated early. However, a delay in diagnosis and management may lead to sequelae associated with gallbladder rupture and biliary peritonitis increasing the mortality rate to up to 5% [2].

## 4 Conclusion

Although rare, it is important to consider GV as a differential diagnosis with acute cholecystitis in an elderly patient. The pre-operative diagnosis of GV is difficult and none of the imaging modalities have proven to be very sensitive. Early intervention can result in rapid resolution thus preventing the potential complication of perforation of the gallbladder into the peritoneal cavity.

## Conflicts of interest

The authors declare that they have no conflict of interest.

## Funding

This study has not received any funding.

## Ethical approval

The study was approved by Ethics Committee of Hospital Sahloul Sousse.

## Consent

Written informed consent was obtained from the patient.

## Author contribution

Study concept or design – MBM, HA.

Data collection – HA, WF, RG.

Data interpretation – MBM, WF, RG.

Literature review – WF, ABA, MM.

Drafting of the paper – HA, YB, SL.

Editing of the paper – MBM, AS, AM.

## Registration of research studies

As this was a case report and not a clinical trial, this study does not require registration.

## Guarantor

Mohamed ben Mabrouk.

Houssem Ammar.

## Provenance and peer review

Not commissioned, externally peer-reviewed

## References

[1] Bhama Anuradha R., Ahari Abdi, Chong Hui Sen (2015). The diagnostic dilemma of gallbladder volvulus: report of a case. Gen. Intern. Med. Clin. Innov..

[2] Tarhan Ömer Ridvan, Barut Ibrahim, Dinelek Hasan (2006). Gallbladder volvulus: review of the literature and report of a case. Turk. J. Gastroenterol..

[3] Matsuhashi Nobuhisa, Satake Shinichi, Yawata Kazunori, Asakawa Eri (2006). Volvulus of the gallbladder diagnosed by ultrasonography, computed tomography, coronal magnetic resonance imaging and magnetic resonance cholangio-pancreatography. World J. Gastroenterol..

[4] Agha R.A., Borrelli M.R., Farwana R., Koshy K., Fowler A., Orgill D.P., For the SCARE Group (2018). The SCARE 2018 statement: updating consensus surgical CAse REport (SCARE) guidelines. Int. J. Surg..

[5] Dayananda Padmike, Praba Ramsh Dhamodaran, Balal Mohommed Rafaideen (2018). Gallbladder volvulus: an uncommon phenomenon: a case report and review of the literature. Clin. Med. Rev. Case Rep..

[6] Pottorf Brian J., Alfaro Leonardo, Hollis Harris W. (2013). A clinician’s guide to the diagnosis and management of gallbladder volvulus. Perm. J..

[7] Mouawad Nicolas J., Crofts Brianne, Streu Rachel, Desrochers Randal, Kimball Beth C. (2011). Acute gallbladder torsion—a continued preoperative diagnostic dilemma. World J. Emerg. Surg..

[8] Reddy Prasanna Kumar, Muralidharan M., Venkatasubramanian R., Yuvaraja S. (2005). Laparoscopic derotation and cholecystectomy for torsion gallbladder. J. Soc. Laparoendosc. Surg..

[9] Nakao A., Matsuda T., Funabiki S., Mori T., Koguchi K., Iwado T., Matsuda K., Takakura N., Isozaki H., Tanaka N. (1999). Gallbladder torsion: case report and review of 245 cases reported in the Japanese literature. J. Hepatobiliary. Surg..

